# A Study on Cause Analysis of Digital Divide among Older People in Korea

**DOI:** 10.3390/ijerph18168586

**Published:** 2021-08-14

**Authors:** Woochun Jun

**Affiliations:** Department of Computer Education, Seoul National University of Education, Seoul 06639, Korea; wocjun@snue.ac.kr; Tel.: +82-2-3475-2504

**Keywords:** digital divide, older people, information literacy, information access, information capability, information utilization

## Abstract

Most people in modern society enjoy various benefits due to the development of information and communication technology and smart technology in modern society. However, due to the digital divide, there is a social class that cannot enjoy abundant benefits. The representative class is the class of older people. In the latest four-year national report on the status of the digital divide, the class with largest digital divide among various information disadvantaged groups, such as the disabled, the low-income class, farmers and fishermen, and older people, is the class of older people. In this study, the causes of the digital divide among older people in Korea are analyzed from various perspectives. To this end, various statistical analyses have been conducted based on national statistical survey works over the past four years. The digital informatization level, which is an index used to measure the digital divide, can be classified into three main components: information access, information capability, and information utilization. Among the three components, the information capability is found to be the lowest. Information capability can also be divided into three components for PCs and mobile devices: installation, use, and management. Among them, management capability was found to be the lowest. Based on this analysis, various plans to reduce the digital divide among older people were proposed.

## 1. Introduction

The rapid development of information and communication technology and smart technology is benefiting most people in modern society. The development of these technologies provides benefits not only for modern individuals but also for various areas in our nation, such as the national economy and national defense. Therefore, basic knowledge and utilization of information and communication technology are becoming essential capabilities for people in the modern society. Furthermore, a lack of understanding of information and communication technologies and the lack of utilization ability can degrade individuals’ quality of life and further reduce national competitiveness. By integrating individuals’ diverse information capabilities, we call them information literacy (also called digital literacy), which is a measure of individual information ability [1,2,3].

This information literacy is becoming an essential ability for every individual in a country. However, there is a so-called digital divide in our society. In other words, there is a social class called the information disadvantaged class, and they do not fully benefit from the knowledge and information society [4,5]. Therefore, national measures are needed for the information disadvantaged class. In Korea, this information disadvantage class includes the disabled, older people, farmers and fishermen, and the low-income class [6]. In this study, older people are defined as those 50 years of age or older [7,8,9,10]. According to a recent four-year study, the group with the largest digital divide is known as the older people class [7,8,9,10].

Information literacy can be categorized and defined in various ways. There are two main types of information literacy in Korea. The first type of information literacy is used in education. The information literacy used in education can be classified into three main categories, that is: information concept, information utilization, and information ethics [11,12,13]. Information concept refers to an understanding of the basic theories and principles of information, and information utilization refers to the ability to solve problems in everyday life using various tools such as a word processor and spreadsheet software. Information ethics also means Internet ethics or information and communication ethics. At the national level, information literacy can be classified as information access, information capability, and information utilization, respectively [6,7,8,9,10]. Detailed definitions of these three categories are covered in Section 2. In this paper, we use information literacy covered at the national level.

According to a recent study analyzing the current status of the digital divide in Korea, the digital divide of older people is the largest among four information disadvantaged classes in Korea. In other words, among the disabled, older people, the low-income class, and the farmers and fishermen, the digital divide of older people was the largest [14]. Therefore, it is becoming a national responsibility to analyze the causes of the digital divide among older people and to improve information literacy among older people.

The purpose of this study is to investigate the causes of the digital divide among older people in Korea. To this end, we analyzed national digital divide statistics over the last four years from 2017 to 2020 [7,8,9,10]. Specifically, the three major elements of the digital divide (information access, information capability, and information utilization) were ranked, and the largest element determining the digital divide was investigated, and the largest element was also classified into three components to rank their importance.

The rest of this paper is organized as follows. In Section 2, we present a theoretical framework. We first introduce the concept of the digital literacy and media literacy, the concept of the digital divide, and also present related previous works. In Section 3, we present the current status and statistical analysis of the digital divide of older people. In Section 4, we present the implication of this statistical results and propose various ways to reduce the digital divide among older people. Finally, in Section 5, we give the conclusions of our work and discuss further research works.

## 2. Theoretical Framework

### 2.1. Digital Literacy and Media Literacy

The use of digital devices is becoming a necessity in everyday life for everyone in the modern knowledge–information society. In addition to access to digital devices, the ability to utilize various digital services and digital tools is becoming a very essential capability. This knowledge of digital as a whole is called digital literacy, and the definitions of academic digital literacy vary considerably. According to Wikipedia, digital literacy refers to an individual’s ability to find, evaluate, and combine clear information while encountering various media on a digital platform [15].

Meanwhile, the media of the 21st century permeates deep into our lives. For people of all ages, including adolescents, the media plays the role of friends and teachers at the same time, becoming a powerful means of accepting and sharing diverse information. We all need to know how to communicate with diverse and new media regardless of our intentions to lead our daily lives, how to critically accept information from the media, and how to share our opinions correctly and legally through the media. Along with the aforementioned digital literacy, media literacy is becoming an essential capability for everyone in the modern knowledge–information society. According to Wikipedia, media literacy encompasses customs that allow people to access, criticize, create, or manipulate the media [16].

Digital literacy and media literacy are inseparable. In other words, digital literacy is bound to include media literacy in that it requires the use of digital media devices and also digital media information. Meanwhile, media literacy is forced to include digital media in that traditional media has shifted from offline media such as newspapers and records to digital information such as web documents. As such, digital literacy and media literacy have similarities, although there are differences. Renee Hobbs presents the similarities and differences between digital literacy and media literacy, as shown in Table 1 below [17].

As shown in Table 1, the overlap between digital literacy and media literacy can be said to be an area where both technical and cultural (or ethical) elements are needed. Digital literacy and media literacy are both essential capabilities in modern society, and each country is strengthening and expanding its education and policies for digital literacy and media literacy [18,19,20].

As previously described, digital literacy and media literacy are essential information capabilities for everyone who lives in a modern knowledge–information society. Therefore, both digital literacy and media literacy are very closely related to the issue of the digital divide. First, cultivating digital literacy ultimately becomes an important means of reducing the digital divide [21]. Similarly, strengthening media literacy education becomes an important way to solve the digital divide problem [22].

### 2.2. Concept of the Digital Divide

In the literature, the digital divide is defined variously, as follows.

According to Wikipedia, the digital divide refers to the gap between those who are able to benefit from the digital age and those who are not [23]. That is, people without access to the Internet and other information and communication technologies will be disadvantaged since they are unable or at least less able to obtain and enjoy digital information and communication between people.

According to Naver, the digital divide refers to the widening gap as access to knowledge and information is unequal between classes, regions, genders, and countries. The digital divide is emerging as a new social and international issue as the gap in access to and use of information, the most important production factor in the information age, is feared to be deepening the income gap [24].

According to Digital Divide Council, the digital divide is “the gap that exists between individuals who have access to modern information and communication technology and those who lack access.” [25]. Additionally, causes of the digital divide include education, income levels, geographical restrictions, motivation and general interest, and digital literacy.

### 2.3. The Measurement of the Digital Divide

Based on the belief that reducing the digital divide can lead to national development through the alleviation of inequality, the Korean government has investigated and announced the digital divide every year since 2000. The Korea Information Society Agency (http://www.nia.or.kr (accessed on 31 July 2021)) is in charge of the survey of the digital divide between the general public and information disadvantaged classes. The agency has published annually the status of the digital divide based on the digital informatization level. The four major information disadvantaged classes include the disabled, older people, farmers and fishermen, and the low-income class.

The digital informatization level, which is a measurement unit of digital divide status in the survey, is a quantitative and comprehensive metric. The digital divide is calculated by the digital informatization level among the general public and the information disadvantaged classes. Specifically, if the digital informatization level of the general public is assumed to be 100, the digital informatization level of information disadvantaged classes to the general public was measured to calculate the difference of the digital informatization level between the general public and the information disadvantaged classes.

The digital informatization level consists of the following specific indices. The number in parentheses represents the relative ratio to 1.

Digital Informatization Level (comprehensive) = Information Access Level (0.2) + Information Capability Level (0.4) + Information Utilization Level (0.4).

① Information Access Level = whether or not they have wired and wireless information devices (0.5) + whether Internet access is always available (0.5).
② Information Capability Level = PC usability (0.5) + mobile device usability (0.5).
③ Information Utilization Level = whether or not they use wired and mobile internet (0.4) + diversity of Internet service use (0.4) + degree of advanced Internet use (0.2).

Table 2 shows the digital informatization level of older people since 2017 [7,8,9,10]. Note that the digital informatization level is the point of older people, assuming that the digital informatization level of the general public is 100.

### 2.4. Previous Works

There have been some research works on the digital divide of older people in Korea.

Park and Kim presented an empirical and technical study of the longitudinal trend of the digital divide change within the older generation [26]. To this end, the study empirically analyzed changes in digital media accessibility, digital media capabilities, and digital media utilization by dividing the response panel of senior citizens aged 55 and older into four more age groups (55–64, 65–74, 75–84, and 85 and older). In particular, in addition to population social factors, they identify the influence of smartphone retention factors, and analyze the relationship between digital accessibility, capability, and utilization. Studies have confirmed that gaps exist within older age groups. They have identified digital media accessibility, which shows a continuous growth trend in all age groups, and found that education and income levels are the most influential factors in smartphone accessibility in all four age groups. Therefore, it suggests that policy consideration and system preparation are necessary. In contrast, changes in digital capabilities and utilization trends have seen relatively significant gaps between 55–74 and 75 years of age and older, and the interaction between smartphone possession and digital capabilities has a positive impact on digital utilization among older adults.

Yim et al. presented a study that intends to focus on the baby boomers and the elderly generation, considering the factors affecting the digital divide gap among the elderly [27]. In particular, they set gender, academic background, household type, usage motive, usage attitude, anxiety about the information intelligence society, life satisfaction, and social support as major factors affecting the digital divide gap among the elderly. To this end, this study analyzed 2300 people aged 55 and over based on data from the 2017 digital divide survey conducted by the Korea Information Society Agency in 2018. As a result of the analysis, baby boomers’ digital engagement was shown to be significantly affected by gender, academic background, usage motive, usage attitude, intelligent information society, and life satisfaction, and the elderly generation was shown to be affected by household type, intelligent society perception, and life satisfaction.

Choi and Choi published a paper aimed at verifying through empirical data whether informatization is a democratic tool for expanding the political participation of the elderly or a problem that strengthens inequality in power resources [28]. In addition, based on the discussion that political behavior is different from generation to generation by internalizing the social and historical context in which individuals grew up, they analyzed whether the relationship between informatization and political participation varies from generation to generation. Data from 225 members of the elderly and 533 of the young and old generations were analyzed from the raw materials for the 2017 Age Integration Survey. According to the analysis, the political participation of the elderly generation is significantly influenced by the use of information, and the political participation of the younger generation is affected statically by information access and information utilization. In addition, the elderly generation confirmed the regulatory effect of the generation, where the increase in information access was not linked to the expansion of political participation, while the youth generation confirmed the increase in information access meaningfully expanded political participation.

Ju et al. presented a study, the purpose of which is to find variables predicting the digital divide of the elderly, analyze gender differences, and use them as basic data for preparing policy directions related to the digital divide of the elderly [29]. To this end, 340 senior citizens (169 males and 171 females) from all over the country who participated in the 2017 Age Integration Survey surveyed nationwide were analyzed. The results of this study are as follows. First, gender differences in the digital divide (information access, information capability, Internet utilization, and mobile utilization) were found to be significantly higher in men, except for the number of devices in information access. Secondly, the variables predicting access (number of devices), the primary digital divide, are educational background, cohabitation with family members, age, and generational integration. Furthermore, educational background, age, and generational integration have been significantly identified as variables for predicting access (usage time). Third, in information capability, which is the secondary digital divide, variables such as the number of devices, gender, educational background, usage time, and living satisfaction were shown to be significant. Fourth, variables such as the number of devices, gender, age, social status, and usage time were significant for the Internet utilization, the secondary digital divide. Fifth, the number of devices, usage time, gender, age, generational integration, economic status, and cohabitation with family members are found to have significant impact ton mobile utilization, the second digital divide.

Hwang analyzed the impact of the digital divide on socioeconomic participation of the elderly by dividing them into baby boomers and pre baby boomers, and based on this, presented policy implications to alleviate the digital divide among the elderly [30]. Based on this, the author presented individual IT education information and unified information services, development and dissemination of customized technologies through research on demand for IT-related policies, analysis of status.

The research works on the digital divide of older people in other countries besides Korea are as follows.

Paul and Stegbauer introduced the status of the digital divide among generations in Germany [31]. In Germany, the proportion of Internet users aged 60 and older is lower than in other age groups. Additionally, older men use the Internet more than older women. In Germany, the proportion of elderly users is gradually increasing, but it falls short of the proportion of young users. They predict that the digital divide between the young and older generations will not be resolved in the near future. In addition, they estimated that education is a major factor in the digital divide of older people.

The medical school of National University of Singapore has released a status report of the digital divide among older Singaporeans. In this report, poor health is the main cause of the digital divide among older people in Singapore. In this report, it was argued that health problems interfere with older people’s Internet use, online communication and social activities between family and friends [32].

Sunkel and Ullmann reported digital divide status for older adults in Latin America including countries such as Chile, Ecuador, El Salvador, Honduras, Mexico, and Peru [33]. Just as the problem of aging has become a global problem many Latin American countries are gradually facing the aging problem. Accordingly, the digital divide in the elderly population is getting worse. Their report shows that there is a significant digital divide not only between countries in Latin America but also within countries. Internet usage has increased in all countries, but their level of use is still very low compared to groups aged 15–29 and 30–59. Additionally, many older people in Latin American countries live in homes with Internet access, but many of them do not use the Internet. This suggests that motivation and skills, namely the desire and ability to utilize information tools, are also necessary. In older adults, a lack of awareness of how these tools can address everyday needs can pose an important barrier to ICT use.

## 3. Statistical Analysis of the Digital Divide of Older People

In this section, we present a statistical analysis on the digital divide of the class of older people in Korea.

### 3.1. The Current Status of the Digital Divide of Older People in Korea

The purpose of this study is to investigate the current status of the digital divide among older people in Korea and also to analyze the causes of the digital divide. In order to achieve this goal, a statistical analysis is conducted based on the digital divide status report published annually by the Korea Information Society Agency since 2000. We conduct a statistical analysis based on the digital divide status report. First of all, we introduce the current status of the digital divide among older people in Korea as follows.

First, Table 3 shows the digital informatization level of four information disadvantaged classes over the four years from 2017 to 2020.

Table 4 shows three elements of the digital informatization level for older people.

As we can see from Table 4, among the three elements of digital informatization level, information capability has the lowest score. Consider the sub elements of information capability, assuming that the information capability is the most important influence of the digital divide of older people. As we discussed earlier, information capability has two elements, PC usability and mobile device usability, respectively. According to [7,8,9,10], PC usability has the following seven sub elements, as follows:

① Installing and deleting software;
② Connecting and using the Internet;
③ Web browser environment settings;
④ Connecting and using a variety of external devices;
⑤ Transferring files over the Internet;
⑥ Detection and treatment of malicious code;
⑦ Creating documents and materials.

The above seven sub elements of PC usability can be categorized as installation, use, and management, respectively. The three categories have sub elements, as follows:

- Installation: ①, ②, ③
- Use: ④, ⑤, ⑦
- Management: ⑥

On the other hand, mobile device usability has the following seven sub elements.

① Basic configuration;
② Wireless network settings;
③ Move files to a computer;
④ Transferring a file to another person;
⑤ Installing and using the required apps;
⑥ Detection and treatment of malicious code;
⑦ Creating documents and materials.

The above seven sub elements of mobile device usability can be categorized as installation, use, and management, respectively. The three categories have sub elements, as follows:

- Installation: ①, ②
- Use: ③, ④, ⑤, ⑦
- Management: ⑥

The following Table 5 shows overall status of the three sub elements of the information capability of older people.

### 3.2. Statistical Analysis of the Digital Divide

The survey data in this study were analyzed using the Statistical Package for the Social Science (SPSS) WIN 25.0 program. One-way ANOVA was conducted to investigate the digital divide of older people in Korea.

(1) Digital Informatization Level of Four Information Disadvantaged Classes

From Table 3, the following analysis results can be obtained for four information disadvantaged classes. Table 6 shows the analysis results for the four classes.

As seen in Table 6, the digital informatization level was the highest among low-income class with 87.78, followed by the disabled with 75.28, farmers and fishermen with 70.63, and older people with the lowest with 63.58, and there was a statistically significant difference (F = 17.00, *p* < 0.001) Therefore, the level of digital informatization is the highest among the low-income class and the lowest among older people.

(2) Digital Informatization Level of Three Elements of Information Capability among Older People

From Table 4, the following analysis results are obtained for digital informatization level of the three elements of information capability among older people, as shown in Table 7.

As seen in Table 7, the average of information access was the highest with 90.85, followed by information utilization with 64.50, information capability with 49.08, and there were statistically significant differences (F = 93.85, *p* < 0.001) Therefore, it can be seen that the level of information access is the highest and the level of information capability is the lowest.

(3) PC Usability

From Table 5, the following results can be obtained for PC usability for older people in Table 8.

According to the PC usability capability for older people, the average of installation was the highest at 1.80, followed use with 1.74, management with 1.64, and there were statistically significant differences (F = 52.05, *p* < 0.05). Therefore, it can be seen that older people have the highest ability to install PCs and the lowest ability to manage PCs.

(4) Mobile Device Usability

From Table 5, the following results can be obtained for mobile device usability of older people in Table 9.

According to older people’s ability to use mobile devices, as shown in Table 9, the average of installation was 2.36, followed by use at 2.08, management at 1.81, and there were statistically significant differences (F = 197.54, *p* < 0.005). Therefore, it can be seen that older people have the highest level in installation capability and the lowest level in management among mobile device usability.

## 4. Discussion

### 4.1. Analysis of the Digital Divide of Older People

In this section, we discuss the causes of the digital divide of older people in Korea based on the analysis results in Section 3, as follows:

As we looked at the current status of the digital information level and statistical analysis in Section 3, the digital informatization level of older people was the lowest compared to that of the general public among the four information disadvantaged classes in Korea. When looking at it in detail, it was found that among the three major factors of digital informatization level, information access, information utilization, and information capability were lowered in order. This means that older people own a variety of PCs and mobile devices and have many opportunities to access wired and wireless communication, but lack the ability to utilize information in their daily life.

Furthermore, we specifically analyzed the information capability that showed the lowest score at the digital informatization level. Information capability is divided into two capabilities: PC usability and mobile device usability. PC usability and mobile device usability consist of three sub elements: installation, use, and management. Statistically, the installation element showed the highest score, followed by the use area and management the lowest area. This is interpreted as the management capability should be of periodic interest and requires continuous updates compared to the first installation and the ability to use it repeatedly once experienced.

In this study, the most direct and quantified data were used to objectively identify the causes of the digital divide among older people in Korea. In other words, the cause of the digital divide was specifically presented with three main factors: information access, information capability, and information utilization. However, factors affecting the digital divide in older people are expected to include a variety of social factors, including health, income and family, in addition to the three factors listed earlier.

The national statistical data of the Korea Information Society Agency, based on this study, presented various survey results and introduced that various factors can affect the digital divide among older people [6,7,8,9,10]. Some of the survey results are as follows. First, it has been found that older men use the Internet more than older women. Second, older people in their 50s were found to have higher utilization rates of information devices and use of Internet services compared to other ages. Third, it was found that the higher the income among older people, the higher the SNS utilization rate and information production. Fourth, older people are more likely to rely on other people’s help than on themselves when they need to solve problems by using information devices.

### 4.2. Improvement Plans for the Digital Divide for Older People

In this section, we propose various plans to reduce the digital divide among older people in Korea as follows.

As discussed in the previous section, the main cause of the digital divide among the three factors of digital informatization level was information capability. In addition, considering the two elements of information capability, PC usability and mobile device usability, the statistical analysis showed that, among the three subcomponents of PC usability and mobile device usability (installation, use, and management), management has the lowest points.

This means that older people must continue to maintain or develop information capability time after time. In addition, a lack of management capability among the three sub elements of information capability also means that management capability must be maintained or developed continuously. In other words, information capability and management capability mean that what is once learned cannot be used continuously.

In order to improve the informatization level of older people, various informatization education opportunities need to be provided above all. In particular, various online education opportunities should be provided in preparation for the further activation of online education after the COVID-19 pandemic has ended, along with offline education. In particular, as the distribution rate of smartphones is ahead of that of PCs, various informatization education contents that can be used for smartphones should be developed and provided. Currently, there are the “50+” site (http://www.50plus.or.kr (accessed on 31 July 2021)), and the “National Informatization Education” site (http://www.itstudy.or.kr (accessed on 31 July 2021)) operated by the Korea Information Society Agency for informatization education of older people in Korea. It is necessary not only to provide educational institutions at the national level but also to provide support for various private informatization education programs.

## 5. Conclusions

In the modern knowledge and informatization society, various information technologies and communication technologies, as well as smart technologies, provide various benefits to most people. Additionally, our lives are becoming better day by day because of these technologies. In order to enjoy these benefits, all people in a modern society must have a certain level of informatization. However, there may be groups that cannot maintain this level of informatization due to physical and mental disabilities as well as economic factors, etc. These groups are called the information disadvantaged classes, and there is a problem with the digital divide in these groups.

This study dealt with the problem of the digital divide among older people in Korea. First, we looked at the current informatization level of the four major information disadvantaged classes. In addition, the current status of the digital divide among older people was examined and the causes of the digital divide among older people were statistically investigated. Based on these analyses, plans were proposed to reduce the digital divide among older people in Korea. To this end, the research was conducted based on the National Informatization Status Report conducted by the Korea Information Society Agency from 2017 to 2020.

In this study, it was statistically shown that the digital informatization level of older people was the largest among the four information disadvantaged classes. It also showed that digital divide of older people comes from the lack of information capability among the three factors of digital informatization level that determine the digital divide. In addition, among the three sub elements of information capability, installation, use and management capability, management capability was vulnerable for older people.

Based on these statistical analyses, we discussed the need for continuous education to bridge the digital divide among older people. In particular, we insist that national education institutions and various private education institutions are needed for older people to access and receive informatization education anytime, anywhere with smartphones.

The limitations of this study are as follows: First, psychological or sociological factors were not considered to objectively analyze the digital divide. Furthermore, more in-depth factors, such as personal interviews, were not considered because national statistics were analyzed as secondary data.

This study introduced the current status of the digital divide among older people in Korea and analyzed the causes of the digital divide and suggested solutions. The question of the digital divide is ultimately to analyze “what causes the digital divide?” The reason why it is important to analyze the cause of the digital divide is that the solution varies depending on the cause. However, as in other countries, the digital divide is characterized by a combination of factors such as society, culture, gender, educational environment and even psychological environment. Therefore, it is very difficult to objectively analyze the causes of the digital divide and it can also change over time and depend on the social environment (e.g., the emergence of COVID-19). Therefore, it is ultimately necessary to develop an objective and accurate analysis method to find the causes of the digital divide in the future.

### Further Research Works

Follow up to this study is as follows. First of all, it is necessary to analyze the causes of the digital divide among older people from various perspectives. In this study, the digital informatization level that could be represented by numerical information was mainly analyzed. However, psychological factors as well as social factors should be analyzed. When combining numerical and non-numerical information, better plans to reduce the digital divide can be drawn up. In addition, various informatization textbooks for older people should be developed. In line with the rapid development of information technology, informatization textbooks should also include up-to-date contents and provide highly readable interfaces and various interactions for older people to see easily.

## Figures and Tables

**Table 1:** Digital Literacy and Media Literacy.

Digital Literacy	Media Literacy
Participates in a collaborative digital knowledge community	Heightened awareness of media use
Uses digital texts, tools and technologies for inquiry learning	Reflects on how media influences attitudes and behaviors
Aware of how digital texts circulate as culture	Aware of how media constructs representations of ideas, events and people in ways that impact democratic processes
Gains competence and confidence with digital technologies by practicing and self-learning	Understands media systems and the political economy of the media
Critically analyzes messages to evaluate credibility and quality	
Creates media for self-expression, communication and advocacy	
Aware of interpretation processes at work in the sharing of meaning	
Balances benefits and risks by using media content in socially responsible ways	

**Table 2:** Digital Informatization Level of Older People by Year.

Year	Digital Informatization Level
2017	58.3
2018	63.1
2019	64.3
2020	68.6

**Table 3:** Digital Informatization Level of Four Classes.

	2017	2018	2019	2020
The Disabled	70.0	74.6	75.2	81.3
Low-Income Class	81.4	86.8	87.8	95.1
Farmers and Fishermen	64.8	69.8	70.6	77.3
Older People	58.3	63.1	64.3	68.6

(Unit: %, Assume that digital informatization level of the general public is 100).

**Table 4:** Digital Informatization Level of Older People by Year.

	2017	2018	2019	2020
Information Access	89.9	90.1	90.6	92.8
Information Capability	41.0	50.0	51.6	53.7
Information Utilization	59.9	62.8	63.9	71.4

(Unit: %, Assume that digital informatization level of the general public is 100).

**Table 5:** Level of 3 Subelements of Information Capability of Older People (4-point Scale).

Usability	Elements	2020	2019	2018	2017
PC Usability	Installation	1.67	1.84	1.82	1.86
	Use	1.62	1.73	1.79	1.81
	Management	1.54	1.6	1.72	1.71
Mobile Device Usability	Installation	2.20	2.39	2.38	2.46
	Use	1.92	2.07	2.12	2.19
	Management	1.68	1.79	1.88	1.89

**Table 6:** Summary of Digital Divide of Four Classes.

	Mean	Standard Deviation	F	*p*
Disabled people	75.28	4.64	17.00 ***	0.000
Low-Income People	87.78	5.63		
Farmers and Fishermen	70.63	5.14		
Older People	63.58	4.24		
Overall	74.31	10.12		

*** *p <* 0.001.

**Table 7:** Summary of Digital Information Level of Information Capability.

	Mean	Standard Deviation	F	*p*
Information Access	90.85	1.33	93.85 ***	0.000
Information Capability	49.08	5.59		
Information Utilization	64.50	4.90		
Overall	68.14	18.44		

*** *p* < 0.001.

**Table 8:** Summary of PC Usability of Information Capability.

	Mean	Standard Deviation	F	*p*
Installation	1.80	0.09	52.05 ***	0.019
Use	1.74	0.09		
Management	1.64	0.09		

*** *p* < 0.05.

**Table 9:** Summary of Mobile Device Usability of Information Capability.

	Mean	Standard Deviation	F	*p*
Installation	2.36	0.11	197.54 ***	0.005
Use	2.08	0.11		
Management	1.81	0.10		

*** *p* < 0.01.

## Data Availability

Publicly available datasets were analyzed in this study. This data can be found here: [http://www.nia.or.kr] (accessed on 13 August 2021).
